# Single-molecule insights into repetitive helicases

**DOI:** 10.1016/j.jbc.2024.107894

**Published:** 2024-10-17

**Authors:** Ya-Mei Zhang, Bo Li, Wen-Qiang Wu

**Affiliations:** School of Nursing and Health, School of Life Sciences, State Key Laboratory of Crop Stress Adaptation and Improvement, Kaifeng Key Laboratory Active Prevention and Nursing of Alzheimer’s Disease, Henan University, Kaifeng, China

**Keywords:** DNA helicase, RNA helicase, single-molecule biophysics, repetitive motion, repetitive function

## Abstract

Helicases are ubiquitous motors involved in almost all aspects of nucleic acid metabolism; therefore, revealing their unwinding behaviors and mechanisms is fundamentally and medically essential. In recent decades, single-molecule applications have revolutionized our ability to study helicases by avoiding the averaging of bulk assays and bridging the knowledge gap between dynamics and structures. This advancement has updated our understanding of the biochemical properties of helicases, such as their rate, directionality, processivity, and step size, while also uncovering unprecedented mechanistic insights. Among these, repetitive motion, a new feature of helicases, is one of the most remarkable discoveries. However, comprehensive reviews and comparisons are still lacking. Consequently, the present review aims to summarize repetitive helicases, compare the repetitive phenomena, and discuss the underlying molecular mechanisms. This review may provide a systematic understanding of repetitive helicases and help understand their cellular functions.

## Helicase families, function, and single-molecule methods

Helicases are motor proteins that track along and unwind nucleic acids, coupling the chemical energy from the hydrolysis of nucleoside triphosphates (1, 2). The first helicase was reported in 1976 from *Escherichia coli* (3). Then, helicases were found to be ubiquitous in all known organisms and involved in nearly all aspects of DNA/RNA metabolism, such as replication, transcription, translation, DNA repair, and telomere maintenance by unwinding duplex or other types of high order DNA/RNA into single strands, displacing DNA/RNA binding proteins, and performing strand annealing (4, 5, 6, 7). Helicases can be classified into six superfamilies (SFs) based on their sequence and domain similarity. SF1 and SF2 are the two largest groups, containing a core of two tandem RecA-like folds, which are typically monomers. Unlike SF1–2, SF3–6 are ring-shaped hexamers (or double hexamers), in which the cores consist of six (or 12) individual RecA-like folds (4, 5). Depending on the type of substrates, helicases can be categorized as either DNA helicases or RNA helicases. According to their unwinding mechanisms, they can be organized as translocases or nontranslocases (8). The latter are nonprocessive and mainly belong to the DEAD RNA helicases of SF2 (9, 10).

Translocases can be characterized by rate, directionality, processivity, and step size. Their unwinding mechanisms can be active or passive (4, 11). Purely active helicases can robustly melt duplex nucleic acids through translocation, with similar speeds for unwinding and translocation, regardless of whether their substrates are GC-rich. However, the unwinding ability of purely passive helicases is insufficient to destabilize duplex nucleic acids directly. Thus, passive helicases stall at the unwinding junction until thermal fluctuations melt the base pairs in front of them. In other words, the unwinding of passive helicases relies on translocating and capturing single strands by using transient thermal fluctuations. Therefore, the unwinding speed of passive helicases is less than their translocation speed on single strands, and their translocation speed is sensitive to GC pairs (deceleration) and external forces (acceleration). Studying the molecular behaviors and mechanisms of helicases *in vitro* is imperative for understanding their biological function *in vivo*. Therefore, in recent decades, many biochemical, biophysical, and structural methods have been used to reveal the mechanisms of helicases (1).

### Single-molecule methods and their advantages

Traditional ensemble biological and biophysical studies using, for example, electrophoresis (12) and fluorescence (13) methods, have determined many fundamental properties of helicases, such as directionality, unwinding ability, substrate preference, and kinetic parameters. However, certain static and dynamic details are averaged in these ensemble-based studies (14, 15), and the exact structural information is lacking. Although structural methods elucidate molecular details through directly capturing intermediate states at the atomic level (4), transient and rare states are difficult to capture. In addition, kinetic information between different intermediate structures is lost in static snapshots (16). Therefore, there is a knowledge gap between dynamics and structures. Single-molecule techniques enable researchers to monitor the real-time dynamic structure of individual molecules, thus bridging these two sides (17).

Using single-molecule technology, researchers can gain the following crucial advantages of revealing the molecular mechanism of helicases (14, 18, 19). First, helicases can be monitored separately in real time; therefore, their functional details such as their translocation and unwinding trajectory and conformational dynamics can be directly captured to access molecular heterogeneity. Second, by moving or immobilizing, the kinetic properties of the same helicase can be studied under different reaction conditions, and even the mechanical properties during the unwinding process can be studied in detail through external force manipulation. Third, single-molecule methods usually have high temporal (millisecond) and spatial (nanometer) resolution. Finally, single-molecule studies only need minute sample amounts (nanomolar (or lower) concentrations and microliter volumes), which is vital when analyzing precious samples.

### Single-molecule techniques for helicase studies

**Figure 1:**
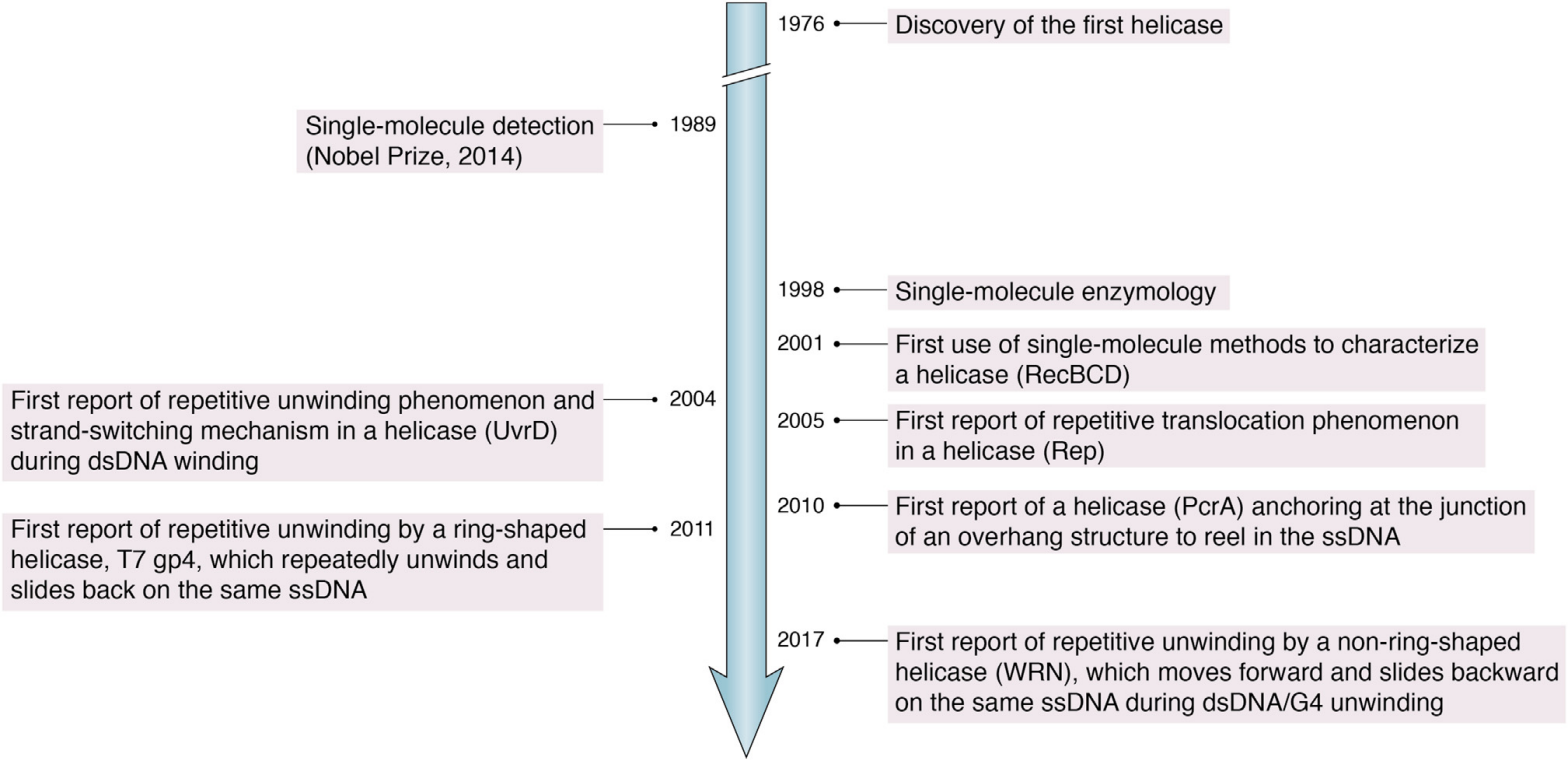
**A brief history of helicase study *via* single-molecule techniques.** 1976, discovery of the first helicase; 1989, detection of the first single molecule; 1998, first application of single-molecule imaging in enzymology; 2001, first application of single-molecule technology to helicases; 2004, discovery of the first repetitive unwinding; 2005, discovery of the first repetitive translocation; 2010, discovery of the first anchoring helicase; 2011, discovery of the first ring-shaped reciprocating helicase; 2017, discovery of the first non-ring-shaped reciprocating helicase.

In 1989, single-molecule detection was achieved by absorption in a solid by Moerner and Kador (20). One year later, in 1990, single-molecule detection was achieved both in solid (21) and solution (22) by fluorescence excitation. In 1998, Xiaoliang Sunney Xie’s lab first monitored the dynamics of individual enzymes using a real-time single-molecule imaging approach (23). In 2001, Stephen Kowalczykowski’s group and Jeff Gelles’ lab first detected the unwinding of the RecBCD helicase at the single-molecule level using optical tweezers-assisted DNA fluorescence imaging (24) and tethered particle motion techniques (25). Since then, increasingly more single-molecule methods have been used to study helicase kinetics (Fig. 1).

**Figure 2:**
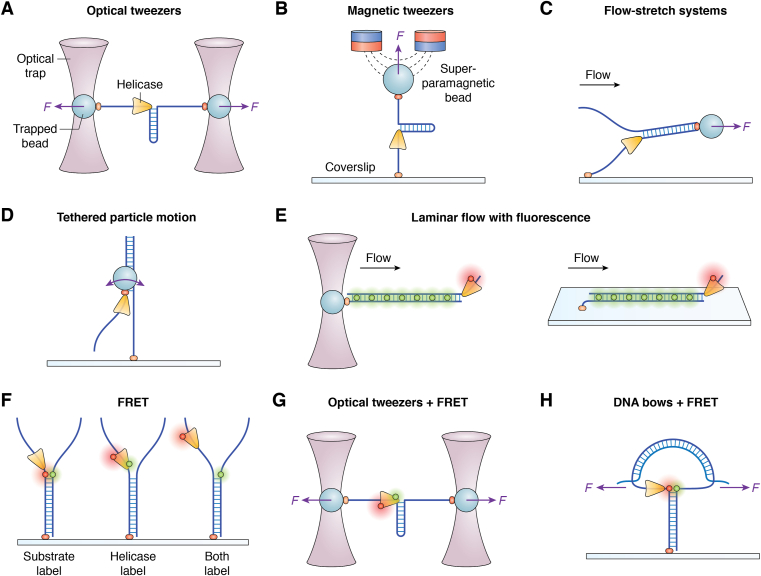
**Schematics of representative single-molecule methods for monitoring the behavior of helicases.** For simplicity, DNA is used as an example. *A*, optical tweezers: this diagram shows dual-trap optical tweezers. A DNA molecule is suspended between two microspheres, with each microsphere held in place by an optical trap. Accurate force is exerted on both microspheres, and changes in the distance between them can be used to infer the transition between double-stranded DNA (dsDNA) and single-stranded DNA (ssDNA). *B*, magnetic tweezers: DNA is attached to both the surface and a paramagnetic microsphere. By applying a magnetic field, the DNA can be stretched with a precise force. Transitions from dsDNA to ssDNA result in a change in the height of the microsphere, which can be used to calculate the length change from dsDNA to ssDNA. *C*, flow-stretch system: similar to magnetic tweezers (*B*), DNA is attached to both the surface and a microsphere. Laminar flow is used to apply a precise force on the microsphere. Conversion from dsDNA to ssDNA leads to an increase in extension length, which is monitored by the position of the bead. *D*, tethered particle motion technique: DNA is immobilized on a coverslip surface, and a microscopic bead is fixed to the other end of the DNA (or to the helicase). By monitoring the Brownian motion amplitude of the microscopic bead, we can determine the length change of DNA (or the position of the helicase). *E*, laminar flow with fluorescence: a dsDNA molecule immobilized on a coverslip or by an optical trap, is labeled with a fluorescent marker and stretched by laminar flow. This setup allows for the direct monitoring of another fluorescently labeled helicase’s movement on the DNA. *F*, single-molecule fluorescence resonance energy transfer (smFRET): by monitoring the transfer efficiency between fluorescent pairs capable of undergoing FRET, changes in distance between them are inferred. The substrate label strategy can be used to monitor the unwinding process of DNA; the helicase label strategy records structural changes of the helicase during unwinding; the both-label strategy monitors interactions between the helicase and DNA. *G*, combined use of optical tweezers and smFRET: this combination allows for the simultaneous recording of the helicase’s structural changes and the process of nucleic acid unwinding. *H*, combined use of DNA bows and smFRET: a nanotensioner is used to prevent thermal fluctuations, resulting in improved spatial resolution.

The types of single-molecule technologies used to study helicases can be divided into three branches: force-based approaches, imaging approaches, and hybrid techniques (Fig. 2). Forced-based approaches use force-based manipulation and spectroscopy, including optical tweezers (Fig. 2*A*), magnetic tweezers (Fig. 2*B*), and flow-stretch systems (Fig. 2*C*). These approaches apply an extra force on a substrate *via* connected beads to manipulate and monitor the behavior of the helicases, as represented in the force-extension traces. The extra force is generated by an optical trap (26), a magnetic trap (27), or fluid flow (28, 29).

Imaging approaches, without application of any extra force, can be used to measure the unwinding and structural dynamics of helicases (19). These approaches include tethered particle motion (30) (Fig. 2*D*), laminar flow with fluorescence (31) (Fig. 2*E*), single-molecule fluorescence resonance energy transfer (smFRET) (32) (Fig. 2*F*), atomic force microscopy (33), and nanopores (34). In tethered particle motion experiments (Fig. 2*D*), researchers can monitor the length of a single DNA molecule in real time by recording the Brownian motion amplitude of a microscopic bead tethered to the end of a single DNA molecule (or one fixed to the helicase) (25). Laminar flow with fluorescence is one of the most common single-molecule approaches, in which both DNA and helicases can be labeled with fluorescent molecules, allowing the direct tracking of helicase motion on DNA (35). The DNA can be immobilized on a coverslip and can also be fixed by an optical trap (24) (Fig. 2*E*). If two fluorescent molecules that can undergo FRET are used in the imaging system, the unwinding process of the nucleic acid (Fig. 2*F*, substrate label), the structural dynamics of the helicase (Fig. 2*F*, helicase label), and the interaction between the helicase and the nucleic acid (Fig. 2*F*, both label) can all be captured using different labeling strategies (32). It should be noted that total internal reflection fluorescence microscopy is usually used for fluorescence correlation imaging in order to obtain a better signal-to-noise ratio (36). Although *in vivo* single-molecule imaging techniques have been used to study the cellular dynamics of helicases (37), they are less effective for understanding microscopic molecular mechanisms in detail. Therefore, this review focuses exclusively on *in vitro* single-molecule techniques. Although atomic force microscopy can serve as a manipulation technique (38), its scanning function has been used more often for helicase unwinding studies (33, 39). Nanopore tweezers can detect the movement of nucleic acids by using the change in the conductance of nucleic acid passing through a nanopore. This technique provides unprecedented spatiotemporal resolution (1 nt and less than 1 ms) (40), but this technique is not universal and has only been used in the study of a limited number of helicases; furthermore, it is challenging to interpret the obtained electrical signals. Generally speaking, different methods have different advantages (reviewed in (41, 42, 43)). For example, smFRET is ideal for studying the details of local structural changes within a distance of 2 to 8 nm; optical tweezers and magnetic tweezes can measure and manipulate long-range and long time frame unwinding.

It is often difficult to delineate the details of the unwinding process from a single perspective using the advantages of a single technique. Therefore, many hybrid techniques were developed. These hybrid techniques include, but are not limited to, integrated fluorescence and optical tweezers (44, 45, 46, 47, 48) (Fig. 2*G*), combined fluorescence and magnetic tweezers (49), and nanotensioners (50) (Fig. 2*H*). Using optical tweezers and FRET hybrid methods, the unwinding behavior and structural dynamics of UvrD were linked (44). A nanotensioner, in which tension from a bent dsDNA molecule is exerted on ssDNA overhangs to prevent their thermal fluctuations, improves spatial resolution to as low as 0.5 bp (50, 51, 52). Because smFRET is easy to understand and can show many details, the smFRET method is used in subsequent examples.

## Repetitive action of helicases

Over the past two decades, single-molecule techniques have been applied to all helicase families and have revealed many remarkable details about their reactions and mechanisms (42, 53). One notable phenomenon is repetitive motion, including repetitive unwinding and repetitive translocation (54). Before studying the behavior of helicases with single-molecule techniques, it was presumed that helicases translocated on nucleic acid substrates unidirectionally before dissociation. This understanding was upended when UvrD (27) and RecBCD (55) helicases exhibited reversal behavior, as determined by using magnetic tweezers (Fig. 1). Later, the Rep helicase was shown to repetitively translocate on the overhang DNA structures (56). Then, an increasing number of helicases, including both non-ring-shaped and ring-shaped helicases belonging to different superfamilies and organisms, were shown to undergo repetitive translocation and unwinding (Table 1), indicating that this is a general property of helicases.

**Table 1:** Helicases exhibiting repetitive action

SF	Helicase		Repetition observed	Mechanism
SF1	UvrD/Rep	EcUvrD	dsDNA unwinding (27, 44)	Switching and translocating back
			Overhang translocation (92)	Anchoring and reeling
		BsPcrA	Overhang translocation (62)	Anchoring and reeling
		EcRep	dsDNA unwinding (108)	Switching and translocating back
			Overhang translocation (56)	Translocation and snapback
		ScSrs2	dsDNA unwinding (109)	-
			Overhang translocation (63)	Anchoring and reeling in reverse
		HsFBH1	Overhang translocation (91)	Translocation and snapback
	Pif1	ScPif1	dsDNA unwinding (65)	Switching and translocating/sliding back
			RNA:DNA hybrid unwinding (78)	Reciprocating
			G4 unfolding (59)	Translocation and snapback
			Overhang translocation (93)	Anchoring and reeling
			ssDNA translocation (98)	Direct looping
		SpPfh1	RNA:DNA hybrid unwinding (78)	Reciprocating
		T4 Dda	dsDNA unwinding (116)	Switching and translocation/sliding back
		DrRecD2	dsDNA unwinding (68)	Switching and translocating back
		HsHELB	dsDNA unwinding (99)	Switching and translocating back
			ssDNA translocation (99)	Direct looping
	Upf1	HsUpf1	dsRNA unwinding (118)	Switching and translocating back
		GsBad	dsDNA unwinding (101)	Reciprocating
		SARS-CoV2 nsp13	dsRNA unwinding (120)	Backward slippage
		ScSen1	dsDNA unwinding (119)	Switching and translocating back
	EcRecBCD		dsDNA unwinding/digesting (55, 117)	-
SF2	RecQ	EcRecQ	dsDNA unwinding (126)	HRDC-dependent shuttling
			dsDNA unwinding (69, 94)	Switching and translocating back
			Overhang translocation (94)	Anchoring and looping
		ScSgs1	dsDNA unwinding (127)	Switching and translocation/sliding back
		HsBLM/RecQ2	dsDNA unwinding (61, 71)	Switching and translocating back (61), Switching and sliding back (71)
			G4 DNA unwinding (86)	-
			Overhang translocation (60)	Anchoring and looping
		CeHIM-6 (CeBLM)	dsDNA unwinding (76)	Reciprocating
		GgWRN	dsDNA unwinding (58)	Reciprocating
			G4 DNA unfolding (58)	Reciprocating
			Overhang translocation (58)	Reciprocating
		HsWRN/RecQ3	dsDNA unwinding (129)	Sliding back
			RNA:DNA hybrid unwinding (66)	Reciprocating
			G4 DNA unwinding (86)	-
		CeWRN-1	dsDNA unwinding (77)	Reciprocating, anchoring and looping
		HsRecQ5β	Overhang translocation (95)	Anchoring and looping
		AtRecQ2	dsDNA unwinding (57)	Switching and translocation/sliding back
		AtRecQ3	dsDNA unwinding (57)	Switching and translocating back
	Rad3	HsFancJ	DNA G4 unfolding (85)	-
		FaXPD	dsDNA unwinding (79, 130)	Backstepping (79), switching and translocating back (130)
	NS3/NPH-II	HCV NS3	dsRNA unwinding (131)	Sliding back or strand switching
			dsDNA unwinding (132)	Snaping or slipping back
			ssDNA translocation (96)	Direct looping
			Overhang translocation (96)	Anchoring and reeling
	Ski2	TgHel308	ssDNA translocation (133)	Backstepping
	RIG-I	HsRIG-I	dsRNA translocation (134)	-
	RecG	EcRecG	HJ branch migration (135)	Strand switching
		T4 UvsW	HJ branch migration (135)	Strand switching
	DEAD	eIF4AI	dsRNA unwinding (89, 90)	Nonprocessive unwinding
	DEAH	HsDHX9	dsRNA unwinding (67)	-
		DHX36	DNA G4 unwinding (86)	-
			RNA G4 unwinding (87)	-
	Cas3	EcCas3	dsDNA unwinding (97)	Anchoring and reeling
SF3	Bovine apillomavirus E1		dsDNA unwinding (74)	Reciprocating
SF4	T7 gp4		dsDNA unwinding (72, 80)	Reciprocating (72) and backstepping (80)
	T4 gp41		dsDNA unwinding (73)	Reciprocating
	SPP1 G40P		dsDNA unwinding (141)	Reciprocating
SF6	9°N MCM		dsDNA unwinding (75)	Reciprocating
	ScCMG		Diffusion on dsDNA (45)	Diffusion
	DmCMG		dsDNA unwinding (102)	Random walking

9°N, *Thermococcus* sp. 9°N; Bs, *Bacillus stearothermophilus*; Dm, *Drosophila melanogaster*; Ec, *Escherichia coli*; Fa, *Ferroplasma acidarmanus*; Gs, *Geobacillus stearothermophilus*; Hs, *Homo sapiens*; Sc, *Saccharomyces cerevisiae*; Sp, *Schizosaccharomyces pombe*; Tg, *Thermococcus gammatolerans*.

### Methods to demonstrate that repeated signals originate from a single helicase

**Figure 3:**
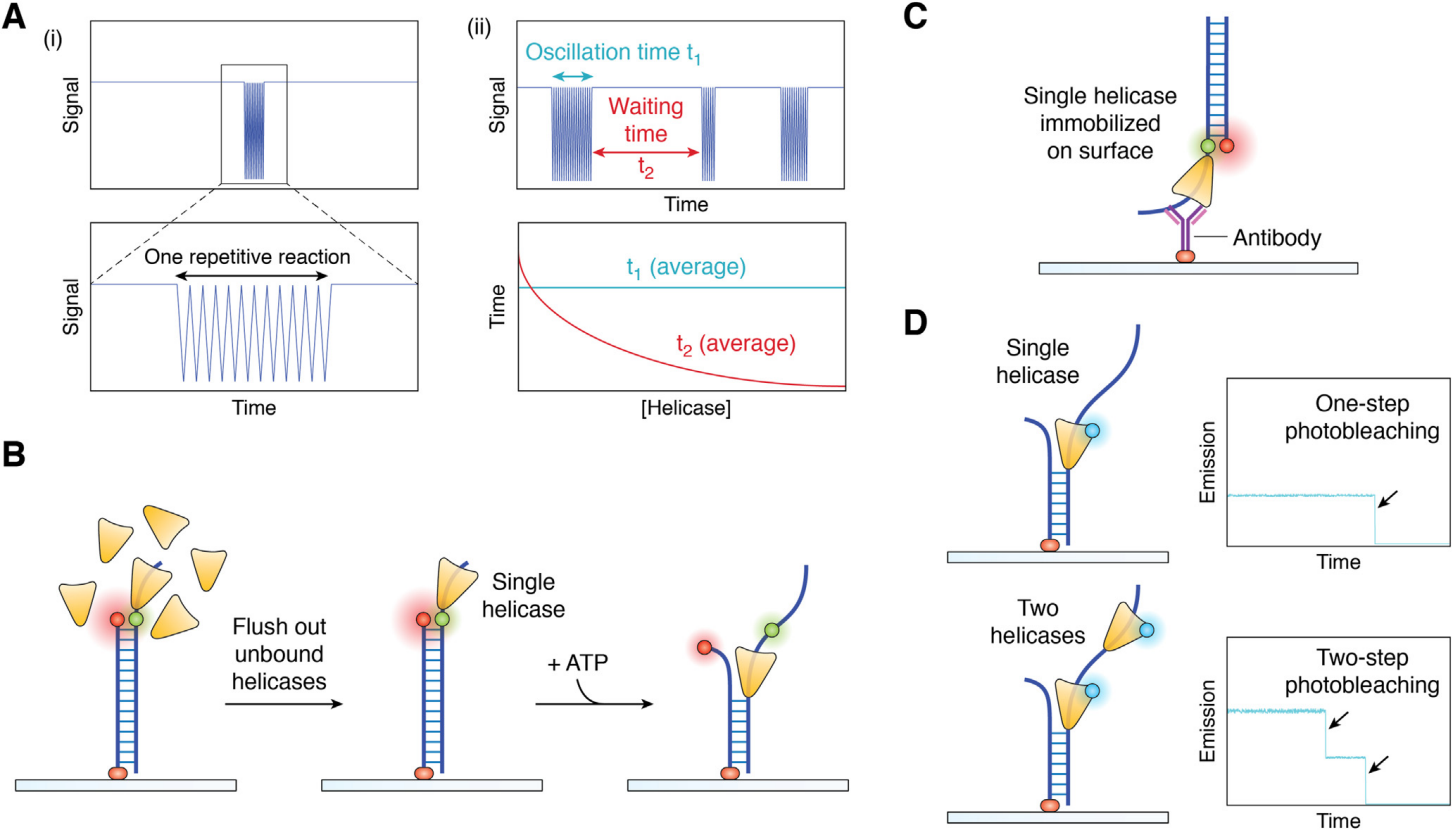
**Methods to confirm repetitive signals resulting from a single helicase.***A*, only one repetitive reaction, including many individual forward and backward motions, occurs over a long period of time (*i*), and the average oscillation time is independent of the helicase concentration (*ii*), while the average wait time decreases with an increasing helicase concentration (*ii*). *B*, free helicase in the solution is washed to ensure that no more helicase binds to the substrate. *C*, antibody-antigen recognition is used to ensure that individual helicases are immobilized on the surface. *D*, the number of helicases is determined by quenching of a single fluorescent molecule labeled on a single protein. In the case of a single helicase, the signal is quenched in one step. If two helicases are bound to a substrate, it exhibits two-step quenching.

A question to be considered is how to distinguish repeated signals from multiple reactions of a single helicase (or stable complex) or successive reactions of multiple helicases. There are four common methods for judging this. First, the concentration of helicases can be changed, which is suitable for all single-molecule methods for helicase study. If it is a repeated reaction of the same helicase, at a sufficiently low concentration, there will be only one repetitive reaction consisting of many individual forward and backward motions over a long period (Fig. 3*A*-(i)) (57). As the protein concentration increases, there is an obvious wait time between the two repeated reactions (58, 59), in which the statistical time of the oscillation has nothing to do with the protein concentration, but the statistical wait time is inversely related to the protein concentration (Fig. 3*A*-(ii)) (60). Second, after coincubating a nucleic acid and one type of helicase, the unbound helicase in solution can be removed by washing to prevent rebinding, and then the reaction can be initiated by adding ATP. If a repetitive phenomenon persists, it may be caused by a single protein (or complex) (Fig. 3*B*) (58, 61). If optical tweezers technology is used, the helicase-bound substrate can also be moved into the reaction solution containing ATP directly. Third, the helicase can be immobilized to prevent successive binding of multiple monomers (or complexes) (Fig. 3*C*) (62, 63). Fourth, photobleaching can be used. A protein can be labeled with a fluorescent molecule; the number of fluorescence quenching steps then represents the number of molecules (Fig. 3*D*) (64, 65, 66). In addition, the total intensity of fluorescence also has potential application (67).

### Repetitive unwinding-reannealing and mechanisms

#### Duplex unwinding by translocases

**Figure 4:**
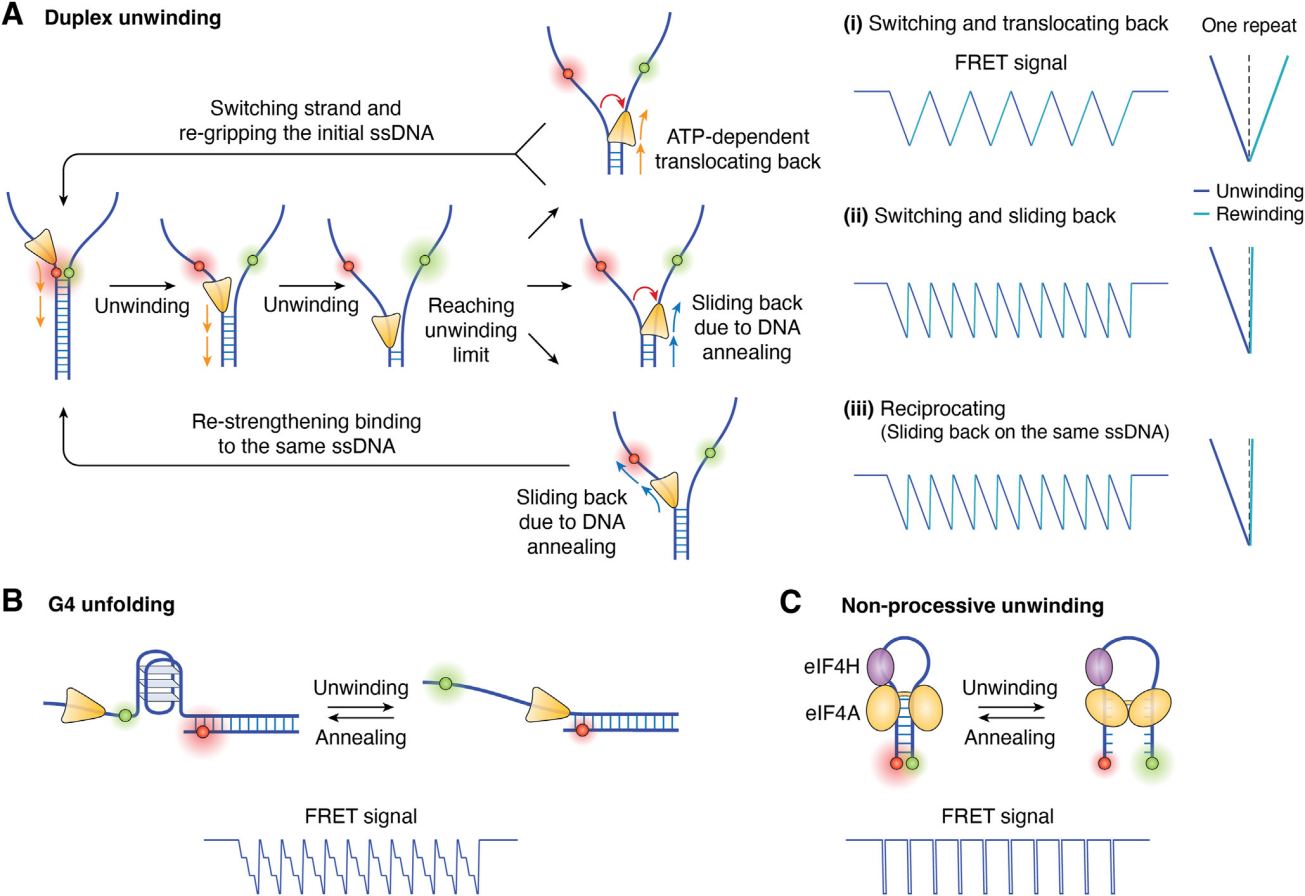
**Schematic diagram of the mechanism and signaling of repetitive unwinding by helicases.***A*, duplex unwinding: when a single helicase reaches the unwinding limit, it does not dissociate from the unwinding fork. Instead, with double-strand annealing, it either translocates back (*i*) to or slides back (*ii*) to the starting point after strand switching, or directly slides back (*iii*) to the starting point without strand switching. *B*, G4 unfolding: after being unwound by a specialized helicase, G4 structures refold, creating conditions for the next round of unwinding. *C*, nonprocessive unwinding: with the help of eIF4H, eIF4A does not dissociate after unwinding the double-stranded RNA, but waits for the RNA to reanneal before starting the next round of unwinding. eIF, eukaryotic initiation factor.

Repetitive actions are often detected during duplex unwinding by translocases (Fig. 4*A*). When one helicase binds and translocates on the ssDNA tail, the forward dsDNA is unwound, showing a gradual increment of the two ssDNA ends. When the unwinding limit is reached, the helicase does not dissociate from the single strand, but moves back to the starting position and begins the next cycle. This cycle of unwinding and returning results in the repetitive unwinding and reannealing of a single helicase on the same substrate, which exhibits a sawtooth pattern. When the helicase reaches the limit of unwinding, depending on the molecular mechanism, there may be three different models:

(1) Switching and translocating back (Fig. 4*A*-(i)). In this model, the helicase switches to the complementary ssDNA strand, translocates backward (DNA reannealing occurs simultaneously), leading to the rehybridization of the duplex, and then switches to the initial strand where it restarts the unwinding process. The feature of one repeat in this model is near symmetric, and the slope is ATP dependent, because the rates of DNA unwinding and reannealing are consistent with the rates of helicase translocation. For purely active helicases, the unwinding signal should be a perfectly symmetric burst, because they exhibit the same rates for both helicase unwinding and translocation. The strand-switching phenomenon was first revealed by studying the unwinding mechanism of UvrD using magnetic tweezers in 2004 (27), and further confirmed by hybrid optical tweezers and smFRET methods (44). Then, many non-ring-shaped helicases were proposed to support this mechanism during unwinding, such as the SF1 helicases DrRecD2 (68) and ScPif1 (65), and SF2 RecQ family helicases (57, 61, 69).
(2) Switching and sliding back (Fig. 4*A*-(ii)). In this scenario, the helicase slides backward on the complementary ssDNA after strand switching, then reinitiates the unwinding cycle through strand switching to the initial strand. One repeat demonstrates a gradual unwinding signal and a more rapid recovery, showing an asymmetric pattern, because the helicase sliding backward should be pushed by reannealing of the melting dsDNA, which is ATP-independent and much faster than unwinding (70). Many helicases adopt this model, including ScPif1 (65), AtRecQ2 (57), and HsBLM (71). Notably, while sliding back, the helicase may also interact with the initial strand (57), and can transfer to a translocating back state (Fig. 4*A*-(i)) while sliding (57, 65).
(3) Reciprocating (sliding back on the same ssDNA, Fig. 4*A*-(iii)). The signal of this model is also asymmetric, similar to the above switching and sliding back model. The difference is that the helicase does not switch strands, but directly slides back on the tracking strand, pushed by duplex reannealing, to show a reciprocating form. The first reciprocating helicase identified was T7 gp4 in 2011, for which slippage was stimulated by ATP (72). The ring-shaped helicases (SF3–SF6) wrap ssDNA and seem unlikely to strand switch; therefore, the repetitive motion of these helicases contributes to this model (72, 73, 74, 75) (Table 1). For some time, researchers thought that only ring-shaped helicases followed this model, and non-ring-shaped helicases typically required strand switching. This understanding was first upended by us in 2017 (58). We found that WRN helicase unwound or translocated repetitively on many types of substrates, and this phenomenon was well explained by reciprocation; this was further confirmed by Hohng’s group using an RNA:DNA hybrid duplex (66). Then, the finding that non-ring-shaped helicases can reciprocate during dsDNA unwinding was confirmed for other RecQ family helicases (76, 77) and Pif1 family helicases (78). Backstepping can also be regarded as a special case of sliding back directly over single base pairs (79, 80).

It is difficult to distinguish between “switching and sliding back” and “reciprocating” because they produce similar unwinding and reannealing signals on a forked duplex (Fig. 4*A*); however, the following clues can be useful. First, ring helicases are unlikely to strand switch. Second, if there is a transition from sliding back to translocating back, it is most likely that strand switching is required (57, 65), because direct sliding back (reciprocating) would result in a new round of unwinding due to regripping and restarting translocation. Third, for translocases that cannot bind RNA, RNA:DNA hybrid substrates can be used to determine where strand switching is necessary (66, 78). For helicases that can bind RNA, certain artificial substrates such as LNA:DNA may be used instead. Fourth, a short hairpin that prevents sliding can be used to confirm the occurrence of strand switching (57, 81). Finally, forked DNA can be replaced with overhanging DNA, which only retains one ssDNA tail needed for unwinding. If the reversed signal can recover to its initial level, we can speculate that the unwinding is a reciprocating model. In the strand-switching model, if the other ssDNA tail is missing, then during the process in which the helicase returns, the helicase can only perform strand switching inside the double strand, resulting in the helicase being unable to restart from its initial position (*i.e.*, on the single strand outside the junction), further causing the signal to fail to return to its starting value (58, 76).

#### G-quadruplex (G4) unwinding

**Figure 5:**
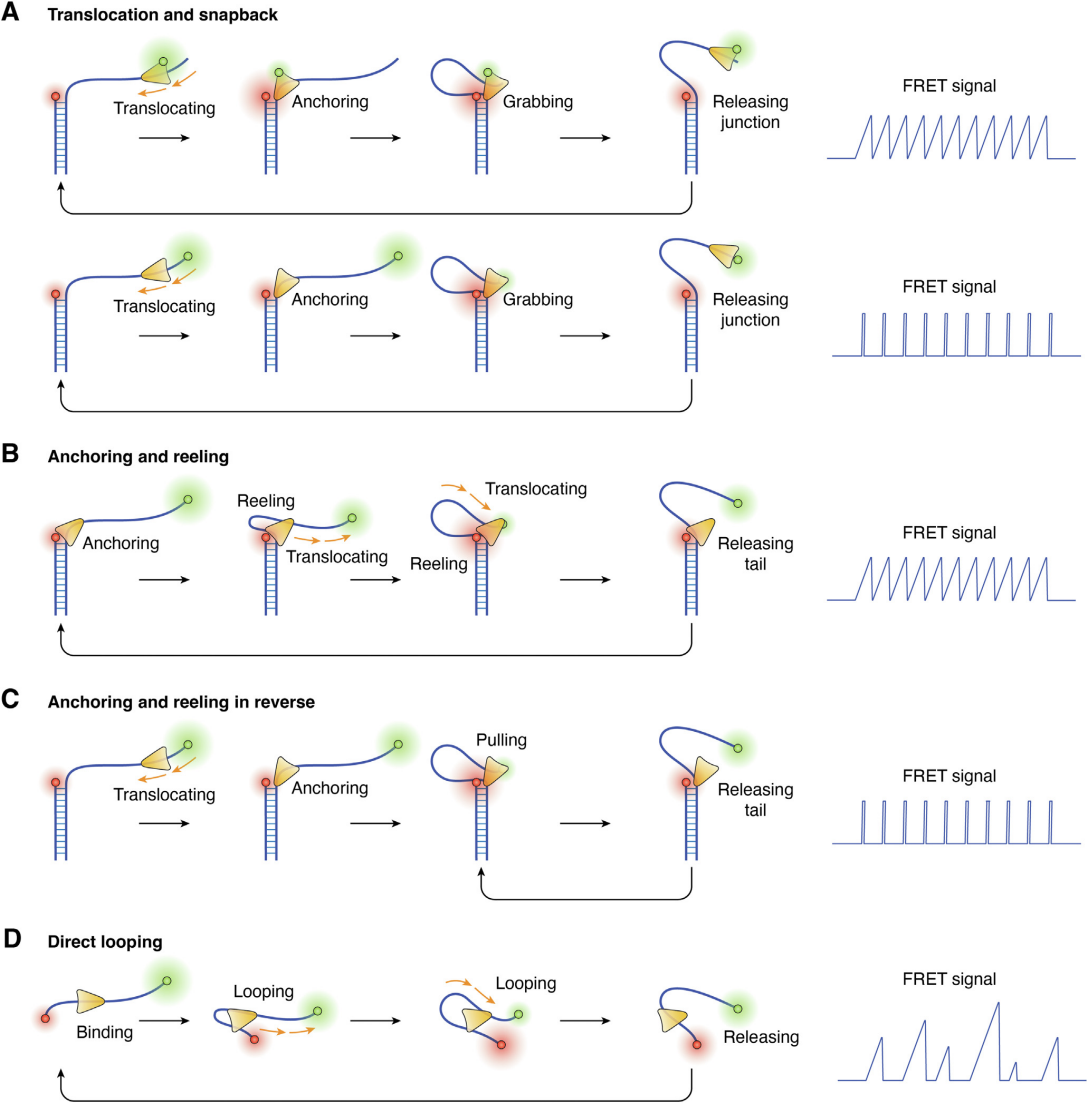
**Schematic diagram illustrating the mechanism and signaling of repeated translocation of helicases.** See the text for details. *A*, translocation and snapback: when certain helicases encounter obstacles during translocation, they regrab the end of the ssDNA and initiate a new round of translocation movement. Different positions of fluorescent labels can present different FRET signals. *B*, anchoring and reeling: a helicase positioned at the junction position repeatedly twitches and releases single strands, showing a gradual increase followed by a sudden decrease in FRET signal. *C*, anchoring and reeling in reverse: unlike the process described in (*B*), here the helicase is anchored at the junction, but it perturbs the single-stranded tail in the direction opposite to its translocation direction. *D*, direct looping: during translocation along a single strand, the helicase occasionally loops and releases the single strand.

G4 structures are non-B-form four-stranded structures folded from guanine (G)-rich nucleic acid sequences (82, 83). G4s can also spontaneously anneal after being unwound, similar to a double strand (84); therefore, G4 unwinding helicases may also show repetitive unwinding (Fig. 4*B*). These helicases include Pif1 (59), FANCJ (85), BLM (86), WRN (58, 86), and DHX36 (86, 87). ScPif1 is proposed to unfold G4 sequentially, hop to the refolded intermediate G-triplex structure, allow complete G4 refolding, and restart the next G4 unfolding cycle (59), similar to tail-rebinding translocation (Fig. 5*A*). GgWRN is proposed to be pushed out of the G4 sequence by refolding G4 (58), similar to the reciprocating model (Fig. 4*A*-(iii)).

#### Nonprocessive unwinding

In addition to translocases, the DEAD family helicases of SF2 are nonprocessive helicases that regulate the conformation of RNA (8) and may disrupt local hydrogen bonds in the form of a “piston” motion (10). As the most representative member of the DEAD family (88), eukaryotic initiation factor, eIF4A1, with the help of eIF4H, unfolds dsRNA in a single step and can repetitively resolve RNA hairpins, which can reanneal after unwinding (89, 90) (Fig. 4*C*).

### Repetitive translocation signals and mechanisms

Repetitive motion is observed not only during the unwinding of duplex and G4 structures, but also during translocation on overhangs and ssDNA (Fig. 5). All these translocases are SF1 and SF2 helicases (Table 1). It should be emphasized that the repetitive translocation referred here does not depend on the higher-order structure of nucleic acids, such as required by duplex and G4 annealing (Fig. 4). Importantly, the monomeric forms of SF1 helicases—such as UvrD family members (UvrD, PcrA, and Rep)—are not processive, despite being highly processive ssDNA translocases and having the ability to efficiently remove single-strand binding proteins (8).

#### Translocation and snapback

In 2005, Ha’s group first reported that the EcRep helicase snapped back to its initial point by rebinding when its translocation was blocked by duplex DNA or streptavidin, and then restarted the next translocation-snapback cycle (56). If two fluorescent molecules are labeled on both the helicase and junction, asymmetrical FRET cycles with a gradually increasing and sudden recovery signal can be observed. If labeled at both ends of a single strand, only the sudden rise and fall of the FRET signal during the snapback process can be observed (Fig. 5*A*). Later, human FBH1 was shown to display similar repetitive shuttling (91), which is reminiscent of the repetitive resolving of G4 DNA by ScPif1 (59).

#### Anchoring and reeling

In 2010, Ha’s group revealed that BsPcrA anchored at the junction of the overhang using a 2B domain and repetitively reeled in the ssDNA tail using two RecA-like domains (62) (Fig. 5*B*). Subsequently, many SF1 and SF2 helicases, including EcUvrD (92), ScPif1 (93), multiple RecQ family helicases (60, 77, 94, 95), and hepatitis C virus (HCV) NS3 (96), were also reported to use this mechanism to perturb ssDNA. Anchoring and reeling is the most common form of repetitive translocation. During repetitive reeling, besides disrupting proteins from ssDNA (62), some helicases also unwind nucleic acid structures (60, 93). In addition, the anchoring position is not necessarily a junction, but may also be another protein complex. For example, Cascade can contact and immobilize the Cas3 helicase, resulting in it reeling and unwinding the dsDNA (97).

#### Anchoring and reeling in reverse

The yeast helicase ScSrs2, the homolog of EcRep, does not snap back to the ssDNA tail when translocating to the dsDNA junction (Fig. 5*A*) but anchors at the junction and repetitively reels in the ssDNA in a direction opposite to its translocation direction (63) (Fig. 5*C*).

#### Direct looping

In recent years, some helicases, including HCV NS3 (96), ScPif1 (98), and human HELB (99), have been shown to loop and release pure ssDNA. The random and repetitive looping show a sawtooth shape with a gradual rise and rapid drop (Fig. 5*D*). This phenomenon implies the existence of additional ssDNA binding sites in addition to the translocation ssDNA binding sites. Interestingly, HCV NS3 has been shown to have a second ssDNA binding site, which has also been confirmed for BLM (100) and bacterial DNA2-like (Bad) helicases (101). Further research is necessary to determine whether other helicases can directly loop.

Above, we have systematically summarized the mechanism of repetitive motion. In addition to the above summary, there are several different mechanisms; for example, ScCMG exhibits random diffusion on dsDNA (45), and DmCMG randomly functions during dsDNA unwinding (102). There are also microscopic mechanisms that are not clear and require more research (Table 1). With additional research efforts, it was found that one helicase had different unwinding mechanisms in different substrate environments (59, 65, 78, 81, 93). Furthermore, repetitive motion on the same substrate may involve a mixture of different mechanisms (60, 61, 77). However, a current problem being researched is how to coordinate and convert different mechanisms.

## Reported repetitive helicases

### SF1

Based on sequence similarity, SF1 can be clustered into three subfamilies, UvrD/Rep-like helicase, Pif1-like helicase, and Upf1-like helicase (103). According to the translocation direction on ssDNA, they can be divided into 3′–5′ SF1A helicases and 5′–3′ SF1B helicases (104). Repetitive helicases have been reported in all three subfamilies (Table 1).

#### UvrD/Rep-like helicases

The monomeric forms of these helicases preferentially translocate in the 3′–5′ direction on ssDNA to disrupt ssDNA binding proteins instead of unwinding dsDNA. The unwinding ability of their monomers is very weak, and therefore the double chain is an obstacle. Their processive unwinding requires dimerization, cofactors, or external forces (8). They were proposed to have two structural conformations, termed open and closed, based on the swivel of their 2B domain (105).

The monomer form of EcUvrD is a poor helicase, exhibiting very low processivity (∼15 bp) for the dsDNA unwinding and dimerization needed for efficient unwinding (44, 46). With the help of an external force from a magnetic trap, monomeric EcUvrD showed repetitive symmetric unwinding-rezipping through strand switching and translocating back (27) (Fig. 4*A*-(i)). It was further revealed that unwinding contributed to the closed conformation of the EcUvrD monomer, and rezipping corresponded to its open formation, as evidenced using integrated fluorescence and optical tweezers (44). Interestingly, UvrD dimers can reduce but not eliminate strand switching (44). Therefore, the poor unwinding ability of EcUvrD results from frequent strand switching. The unwinding and rewinding of dsDNA have the same frequency (27, 44), but this process can be disrupted by the sliding clamp EcMutL, which enhances its processivity (106). In addition to repetitive unwinding on a fork substrate, during translocation, UvrD prefers loading at the 5′-ss-duplex DNA junction and repetitively reels in ssDNA, inducing a small loop (Fig. 5*B*), and then initiates translocation from the junction (92).

BsPcrA can anchor at the junction of the 5′ overhang (or fork) and repetitively reel in and release 3′–5′ ssDNA in an open conformation (Fig. 5*B*) (62). Through this clear translocation cycle, many properties, such as the translocation rate and step size, can be directly obtained, thus avoiding static disorder (62). During the looping, PcrA can efficiently remove RecA filaments (62), but cannot resolve G4 DNA (93).

EcRep translocates on ssDNA in the 3′–5′ direction in an open form. Using smFRET, it was found that when EcRep faces a block, such as dsDNA, it will change to a closed form, snap back to the 3′ end, and restart the next cycle translocation (56) (Fig. 5*A*). Combining the structural dynamics of UvrD and PcrA, it is not difficult to conclude that the open form of UvrD/Rep-like helicases corresponds to translocation, and the closed form corresponds to unwinding. This is why when the structures of Rep and PcrA are fixed in a closed conformation through intramolecular cross-linking, their monomers will transform into processive superhelicases (107). Recently, the 2B-deficient EcRep (RepΔ2B) and WT EcRep were shown to repetitively unwind dsDNA by the strand-switching and translocating back mechanism using optical tweezers (108), highlighting a more complex role for the 2B. Unlike EcRep, ScSrs2 was found to bind at the junction and pull the ssDNA in reverse when encountering a dsDNA block (Fig. 5*C*), with its repetitive motion limited to ∼20 nucleotides (63). In addition, ScSrs2 was reported to repetitively unfold trinucleotide repeat dsDNA hairpins (109). The human F-Box DNA helicase FBH1, a functional ortholog of ScSrs2 (110), uses the same mechanism as EcRep to translocate on 3′ overhangs (91). The repetitive translocation of Rep, Srs2, and FBH1 can remove and prevent DNA recombination proteins RecA/Rad51 to bind ssDNA.

#### Pif1-like helicases

Pif1-like helicases translocate in the 5′–3′ direction on ssDNA. Of them, ScPif1 is one of the well-studied helicases. The monomer form of ScPif1 has a weak dsDNA unwinding ability, and dimerization is required for efficient unwinding (111, 112), similar to UvrD/Rep-like helicases. However, the monomer shows robust G4 DNA and high processive RNA:DNA resolving abilities (113, 114). Monomeric ScPif1 was shown to anchor itself to the junction of 3′ overhangs and fork structures, and periodically reel in the 3′ tail (Fig. 5*B*) (93). During the reeling, it can efficiently unwind RNA:DNA hybrid structures and G4 DNA. Interestingly, this patrolling still exists under external forces (98). ScPif1 does not need to bind the junction and can directly loop ssDNA during ssDNA translocation (Fig. 5*D*) (98). ScPif1 unwinding of G4 DNA (59), dsDNA (65), and RNA:DNA hybrid structures (78) also exhibits repetitive motion. For 5′-ss-G4-dsDNA unwinding, monomer ScPif1 sequentially resolves G4 to ssDNA, halts at the junction, then snaps back to the refolded G4 and restarts the next cycle of unfolding (Fig. 4*B*) (59), similar to the repetitive shuttling of EcRep (Fig. 5*A*) (56). In addition to repetitively reeling the 5′–3′ ssDNA at the fork (93), ScPif1 can also repetitively unwind forked DNA with very limited processivity (less than 10 bp) showing slow unwinding and fast rezipping (65). External forces can enhance the unwinding rate and processivity. Under higher forces, slow rezipping is present. The slow rezipping rate is almost the same as the unwinding rate. Therefore, ScPif1 was proposed to repetitively resolve dsDNA through a switching and translocating back (Fig. 4*A*-(i)) or sliding back (Fig. 4*A*-(ii)) mechanism (65). Later, both ScPif1 and SpPfh1 were shown to unfold RNA:DNA hybrid structures repetitively (78). Therefore, it was concluded that the repetitive unfolding of Pif1 was independent of strand switching, because ScPif1 cannot bind ssRNA (115). Moreover, directly sliding back should also be present in dsDNA repetitive unwinding (65, 78), and whether strand switching actually exists during dsDNA unwinding is challenged. Strand switching is indeed a native property of ScPif1, which can be induced by obstacles such as G4 DNA, LNA:DNA, and RNA:DNA hybrid structures (81). Thus, the mechanism of ScPif1 unwinding dsDNA is very complex, and it is possible that these different mechanisms are also combined. Further in-depth study is necessary to determine how the different mechanisms are coordinated.

The monomer form of the Pif1-like helicase T4 phage Dda (116), DrRecD2 (68), and HsHELB (human DNA helicase B) (99) also show repetitive motion. T4 Dda unwinds dsDNA repetitively, using switching and translocation or a sliding back mechanism, at forces higher than 8 pN (116). DrRecD2 shows a near symmetric dsDNA unwinding pattern, utilizing a switching and translocating back mechanism, revealed by both smFRET and magnetic tweezers (Fig. 4*A*-(i)) (68). HsHELB can unwind dsDNA repetitively and shows a symmetric unwinding pattern (Fig. 4*A*-(i)). It can also directly loop ssDNA, showing an asymmetric pattern (Fig. 5*D*) (99). RecBCD is an enzyme complex composed of three subunits: RecB, RecC, and RecD. Except for RecC, both RecB and RecD subunits are helicases. RecB is an UvrD/Rep-like helicase, while RecD is a Pif1-like helicase. EcRecBCD exhibits backward motion during dsDNA unwinding (55, 117).

#### Upf1-like helicases

Upf1-like helicases are DNA and RNA helicases, belonging to the 5′–3′ SF1B family (103). Human Upf1 performs strand-switching and backward translocation during dsRNA unwinding (Fig. 4*A*-(i)) (118). In addition, ScSen1 also undergoes cyclical translocation and switching (119). SARS-CoV-2 nsp13 exhibits repetitive motion during dsRNA unwinding, showing backward slippage (Fig. 4*A*-(iii)) (120). Moreover, a bacterial DNA2-like (Bad) from *Geobacillus stearothermophilus* backslides directly during dsDNA unwinding through a reciprocating mechanism (101).

### SF2

SF2 helicases, which are involved in almost all aspects of RNA metabolism and many aspects of DNA metabolism, are the largest and most diverse SF classed into 10 subfamilies (103, 121): RecQ-like, Rad3/XPD, Ski2-like, retinoic acid inducible-gene I (RIG-I)-like, NS3/NPH-II, RecG-like, DEAD, DEAH, type I restriction enzyme, and Swi/Snf families. At present, most subfamilies are known to have members that exhibit repetitive motion.

#### RecQ-like helicases

Due to their functional importance, RecQ-like family helicases are among the most studied helicases (6), which translocate along 3′–5′ ssDNA and are conserved from bacteria to humans. In humans, there are five active RecQ-like helicases, HsRecQ1, HsRecQ2/BLM, HsRecQ3/WRN, HsRecQ4/RTS, and HsRecQ5β, of which mutations in the first four will result in RECON (122), Bloom (123), Werner (124), and Rothmund–Thomson (125) syndromes, respectively. Currently, RecQ helicases from many species, including bacteria, yeast, worms, vertebrates, and even plants have been reported to share repetitive motion, indicating that this is a natural property of RecQ helicases.

EcRecQ helicase unwinds dsDNA showing repetitive movement behavior (69, 94, 126). Kovács’s group used magnetic tweezers to reveal that EcRecQ unwound both hairpin DNA and gapped DNA, representing pausing and short-extent shutting (repetitive unwinding-reannealing) behavior (126). The reannealing events included two types, one slow and the other rapid. The slow reannealing resulted from strand switching, which was independent of the HRDC domain, as this phenomenon was observed in all RecQ variants, including a HRDC deletion construct (126). The rapid short-extent reannealing is mediated by the HRDC domain binding to displaced ssDNA (126). Using similar EcRecQ and mutants, Croquette’s group used longer DNA hairpins to study the behaviors and mechanisms. It was found that EcRecQ exhibited strand-switching behavior both during the unwinding process and when arriving at the hairpin apex, which is also independent of HRDC domain (69). The strand-switching behavior of EcRecQ was further confirmed by Xi’s group using smFRET forked dsDNA without an external force (94). The low unwinding processivity of EcRecQ is attributed to repetitive unwinding, which is promoted by the HRDC domain. Thus, HRDC suppresses the duplex unwinding of EcRecQ (94). In addition to duplex unwinding, EcRecQ also shows repetitive translocation on the 5′ ssDNA overhang due to anchoring and reeling (94).

ScSgs1 undergoes repetitive motion during dsDNA unwinding. Each cycle includes gradual unwinding and abrupt or gradual reannealing, with a sawtooth-like pattern (127), indicating the use of a switching and translocating or sliding back mechanism. Interestingly, the repetitive unwinding and rewinding pattern can be modulated by its interaction proteins. In the presence of ssDNA-binding protein ScRPA and ScSgs1, no rapid DNA rezipping was detected; only gradual reannealing occurred (127). The rewinding rate was independent of external force, indicating that the gradual re-annealing resulted from Sgs1 modulation by replication protein A (RPA), not from the passive dissociation of RPA, which was force-dependent (128). In addition, the physical interaction of ScDna2 with ScSgs1 can limit the backward movement of ScSgs1, resulting in the disappearance of full rewinding, and alleviating the inhibition of RPA on Sgs1 unwinding and rewinding velocities (127). Furthermore, Top3 (DNA topoisomerase 3), Rmi1 (RecQ-mediated genome instability protein 1) and Sgs1 can form functional complexes in cells. ScTop3-Rmi1 can stimulate the unwinding velocity of ScSgs1, and reduce the fraction of partial rewinding, which typically involves rapid rezipping. In the presence of ScRPA and ScTop3-Rmi1, ScSgs1 unwinds dsDNA gradually, followed by only gradual DNA rewinding (127). Overall, these results demonstrate the complexity of the regulation of repetitive ScSgs1 by interacting proteins.

In 2009, HsBLM was the first reported RecQ family helicase with repetitive behavior (61). It was shown that once HsBLM unwound a certain length of duplex DNA, it would switch to the opposite strand, translocate back, and restart the next cycle of unwinding (Fig. 4*A*-(i)), exhibiting a repetitive manner regardless of the presence of RPA. Later, HsBLM was proposed to tend to slide back rather than translocate back after strand switching, as demonstrated using magnetic tweezers (Fig. 4*A*-(ii)). In this mode, the RQC domain plays a key role in both forward unwinding and backward sliding by capturing both the dsDNA and 5′ tail, and the amino acid K1125 in the RQC domain may be involved in sliding (71). In addition to duplex unwinding, HsBLM can also unwind G4 DNA repetitively; however, the underlying mechanism is unknown (86). HsBLM can also anchor at the ss/dsDNA junction, reel the 5′ ssDNA tail, and resolve G4 DNA (60). Because reeling can also happen on the fork structure, we revisited the repetitive behavior of HsBLM during dsDNA unwinding and proposed that the repetitive signals should include ssDNA reeling (60). This situation of mixing repetitive unwinding-reannealing and repetitive translocation represented in oscillating signals was recently demonstrated in a study of CeWRN-1, by labeling different substrates and different positions with fluorescence (77). CeHIM-6 (CeBLM) was found to reciprocate on the same strand (Fig. 4*A*-(iii)) (76). Thus, it is possible that BLM may not need to strand switch during a repetitive unwinding-reannealing cycle.

The GgWRN helicase exhibits repetitive motion, including dsDNA unwinding, G4 DNA unwinding, and overhanging DNA translation, with frequent sliding. These repetitive behaviors can be well interpreted by GgWRN reciprocating on the same ssDNA, but not by strand switching (58). The HsWRN helicase repetitively unwinds forked DNA with gradual unwinding and one-step rewinding (129), which likely results from direct back sliding (reciprocating), as replacing the complementary strand with ssRNA still allows for repetitive unwinding, indicating that HsWRN does not need to bind the complementary strand (66). In addition, HsWRN is able to strand switch, because in the presence of a high concentration of RPA, a gradual rewinding phase appears, indicating that HsWRN translocates backward after strand switching to the complementary strand (129). It should be noted that this RPA-modulated rewinding phase change was also found in ScSgs1 (127), indicating that this may be a common property of RecQ helicases. In addition to duplex DNA, HsWRN was also shown to repetitively resolve G4 DNA (86).

RecQ helicases are conserved in plants. The unwinding mechanisms of RecQ2 and RecQ3 helicases from the model plant *Arabidopsis* have been studied by magnetic tweezers. It was revealed that both AtRecQ2 and AtRecQ3 showed repetitive unwinding, while their mechanisms are different (57). AtRecQ2 primarily slides back and translocates back as an auxiliary process after strand switching upon reaching its unwinding limit. In contrast, AtRecQ3 only translocates backward after strand switching. The different unwinding mechanisms within the same family of helicases in the same species may explain why different RecQ helicases have different biological activities (57). Other RecQ family helicases also show repetitive motion; for example, HsRecQ5β can repetitively reel the 5′ ssDNA overhang by using an anchoring-looping mechanism (95).

With additional research efforts, the mechanisms behind the repetitive phenomena of RecQ family helicases have been gradually revealed. This may be the result of a combination of multiple mechanisms. Distinguishing and confirming these mechanisms still requires systematic research and comparisons.

#### Other SF2 helicases

In addition to the RecQ family helicases, there are also other SF2 helicases that behave repetitively. The Rad3/XPD helicase HsFancJ can repetitively unfold G4 DNA (85), although the underlying mechanism is unknown. FaXPD unwinds dsDNA hairpins, with gradual unwinding and sudden backward rezipping using a stepping and backstepping/slipping mechanism (79). Later, in subsequent studies under the same system, FaXPD showed sliding back in low-processivity bursts and translocating back in high-processivity bursts, and the fraction of high-processivity bursts increased with higher concentrations of replication protein A 2 (RPA2) (130). This change in helicase repetitive behavior resulting from RPA interactions has been monitored in ScSgS1 (127) and HsWRN (129). The NS3/NPH-II family helicase HCV NS3 is one of most studied model helicases. It can repetitively unfold both dsRNA (131) and dsDNA (132). Using optical tweezers, Bustamante’s group found that during dsRNA unwinding, HCV NS3 rarely slips back or translocates back (131). Using smFRET, it was found by Ha’s group that HCV NS3 exhibits repetitive dsDNA unwinding behavior using either snapping or slipping back methods when encountering obstacles (132). Recently, Ha’s group showed that the helicase domain of NS3 (NS3h, 450 amino acids) can repetitively loop the ssDNA directly during translocation or by anchoring at the junction (96). This phenomenon caused researchers to reconsider whether other helicases also directly loop ssDNA during translocation. Using nanopore tweezers, it was found that the Ski2-like family helicase TgHel308 exhibited frequent forward movements and backward retreats (133). The RIG-I family helicase HsRIG-I can repetitively translocate on both dsRNA and RNA:DNA heteroduplexes (134). RecG-like helicases, including EcRecG and T4 UvsW, can strand switch during dsDNA unwinding, thus switching in the direction of the Holliday junction branch migration (135). DEAH family helicases, such as DHX9 and DHX36, also show repetitive motion. DHX9 (RNA helicase A) can repetitively unfold dsRNA, and the repetitive manner is dependent on its N-terminal dsRNA binding domain (67). DHX36 (RHAU) was shown to repetitively resolve both G4 DNA (86) and G4 RNA (87). The partial repetitive unfolding-refolding activity is independent of ATP for G4 DNA (86, 136), while for G4 RNA, DHX36 shows ATP-independent unfolding and ATP-dependent refolding (87). Cas3 helicases, which play important roles in the CRISPR-mediated adaptive immune systems, are a new group of SF2 helicases that are evolutionarily close to DEAH and NS3/NPH-II families (121). The cascade complex can immobilize Cas3, causing Cas3 to reel and unwind dsDNA (97). Thus, anchoring is not necessarily at the junction, and may be to another protein complex. In addition to translocases, the nonprocessive DEAD helicase eIF4AI, in conjunction with eIF4H, can bind loop structures and repetitively unfold dsRNA (90). The repetitive folding-unfolding cycle is shown to result from the conformational transition of eIF4AI between an “open” and a “closed” conformation (89).

### Replicative helicase (SF3, SF4, and SF6)

SF3–6 helicases are ring-shaped hexameric helicases. Except for transcription termination factor Rho of SF5, the other three superfamilies are involved in DNA replication (137) by encircling the leading strand and moving along ssDNA. SF3 is composed of eukaryotic virus replication helicases. SF4 comprises prokaryotic replication helicases. SF6 comprises replicative minichromosome maintenance (MCM) helicases from eukaryotes and archaea. In eukaryotes, MCM helicases are heterohexamers (MCM2–7). However, archaeal MCM helicases are homohexamers in most archaeal species (138). Because replicative helicases are circular, they are unlikely to perform frequent strand switching, and therefore most of them may use the reciprocating mechanism (Fig. 4*A*-(iii)). Remarkably, T7 gp4 can strand switch occasionally, but it cannot perform the next round of unwinding, and therefore cannot repetitively strand switch (139). Single-molecule techniques have been widely used to study the dynamics of replicative helicases (140). *Bovine papillomavirus* E1 helicase (74), T7 phage gp4 (72), T4 phage gp41 (73), *Bacillus subtilis* bacteriophage SPP1 G40P (141), and *Thermococcus* sp. 9°N MCM (75) have been shown to repetitively unwind dsDNA using a reciprocating mechanism. In eukaryotes, MCM occurs as the Cdc45-MCM-GINS (CMG) complex. It was shown that ScCMG diffuses rapidly on dsDNA to find a replication fork to start replication (45). In addition, DmCMG unwinds dsDNA repetitively exhibiting random walking that includes unwinding, annealing, and pausing, but not unidirectional motion (102).

## Potential functions of reiterative motion

Due to technical limitations, it is still unclear whether repetitive motion exists *in vivo*. Given that many helicases display repetitive behaviors, it can be concluded that this is an inherent—and not accidental—property. The potential significances of repetitive motion may include the following four aspects. First, repeated motion is very economical for cells, which do not need to express multiple proteins to function. Second, this repetitive behavior allows the enzyme to work uninterrupted, keeping the interacting nucleic acids or proteins in a relatively stable state, and thus promoting the continuous and efficient progress of downstream reactions. For example, the repetitive unwinding of some helicases, such as HsBLM (71) and GgWRN (58), can maintain dsDNA or G4s in an open state, potentially aiding polymerases, telomerases, and other enzymes involved in nucleic acid metabolism. Additionally, the repetitive translocation of some helicases, such as ScSrs2 (63), BsPcrA (62), and HsFBH1 (142), can remove proteins such as Rad51 and RecA from ssDNA, and prevent their rebinding and deactivating unwanted reactions. Third, this behavior may help the helicase find the correct location to function in cells; for example, the diffusion of ScCMG is important for it to find and enter a replication fork (45). Finally, the repetition may be a response of the helicases to specific structures. For example, ScPif1 (81) and HCV NS3 (132) display specific repetitive signals when encountering obstacles.

## Discussion

Before single-molecule intervention in the study of helicase kinetics, it was difficult to imagine that a single helicase can exhibit repetitive motion (Fig. 1). The repetitive behaviors illustrate that the low processivity of SF1 and SF2 helicases is not from complete dissociation after a single cycle of activity. Except for the SF5 transcription termination factor Rho helicase, the other five superfamilies have all been shown to include helicases that can undergo repetitive motion, including unwinding and translocation (Table 1). Furthermore, the repetition can be modulated by the surrounding environment, such as interacting proteins (127, 129), nucleic acid structures (98), external forces (65), and the energy supply status (58, 72), further strengthening the evidence for its existence in cells. Other non-helicase nucleic acid-interacting proteins, such as SSB (143), RPA (144), RAD51 (31), and RecA (145), also exhibit back-and-forth activity, suggesting that this phenomenon may be widespread in the interaction between proteins and nucleic acids.

Although repetitive motion appears to be an intrinsic property of helicases, it has not received attention as a basic property of helicases. The mechanisms of some helicases that exhibit repetitive motion have not been revealed (Table 1). Determining the mechanism of repetitive motion through structural methods, especially understanding the structural basis of the key points that repeatedly initiate the next round, and verifying these findings through single-molecule methods will greatly improve our understanding of the repetitive phenomenon in the future. Understanding the underlying mechanism of the repetition *in vitro* is beneficial for predicting the *in vivo* helicase behaviors and comprehending its biological functions. In addition, this periodic repetitive signaling and regulation is likely to find widespread use in biosensing. At present, a variety of repetitive unwinding and translocation models have been proposed (Figs. 4 and 5); however, our understanding of the mechanism of helicase repetitive movements is far from sufficient.

Different single-molecule techniques reveal different aspects of the underlying mechanism in the repetitive process of helicases. For the extensively studied Pif1 and RecQ family proteins, it is found that multiple mechanisms may be involved in repetitive motion, and mixed looping cannot be ruled out. It is unclear when the same helicases, such as ScPif1 (65, 93) and CeWRN-1 (77), choose to unwind or translocate on the same substrate. In the processes of unwinding and translocation, the following questions are raised: which mechanism does the helicase choose? What factors regulate these processes? Some clues have been obtained on these issues. It was found that in repetition, forward unwinding and backward rewinding may be regulated by the configuration of the helicase (44). Repetitive mechanisms may be modulated by interacting proteins and forces; for example, RPA (127, 129, 130) and an external force (65) can switch helicases from sliding back to translocating back. The repetition can also be modulated by obstacles (81). Helicases of the same family share similar sequences, but they may exhibit different repetitive behaviors on the same substrate (57, 68). It will be interesting to further reveal the mechanisms of helicases from the structural and single-molecule levels, realize the regulation of their behavior, and then apply this to cells.

Directly monitoring the detailed kinetics of helicases within cells is one of the key purposes of research. Although recent developments in imaging technology have enabled the real-time monitoring of other motor proteins, such as kinesin using MINFLUX (146, 147) with high temporal and spatial resolution, monitoring the dynamics of a single helicase within cells remains a challenge and an area for future development. Currently, mimicking the cellular environment in a cell-free system (148) may be a feasible alternative.

## Conflict of interest

The authors declare that they have no conflicts of interest with the contents of this article.
